# Application and progress of palliative therapy in advanced gastric carcinomas

**DOI:** 10.3389/fonc.2023.1104447

**Published:** 2023-03-09

**Authors:** Xiao-fan Luo, Ye-hao Luo, Xin-yi Zhao, Xue-ting Lin, Wen-ling Li, Jie Jie, Di Wu, Gang Fang, Yu-zhou Pang, An Huang

**Affiliations:** ^1^ Key Laboratory of Basic and Applied Research of Zhuang Medicine Prescriptions, Guangxi University of Traditional Chinese Medicine, Nanning, Guangxi, China; ^2^ School of Second Clinical Medicine, Guangzhou University of Chinese Medicine, Guangzhou, China; ^3^ Zhuang Yao Pharmaceutical Engineering and Technology Research Center, Guangxi University of Traditional Chinese Medicine, Nanning, Guangxi, China

**Keywords:** gastric carcinomas, palliative therapy, application, treatment planning, research progress

## Abstract

Gastric carcinomas have high morbidity and mortality. It produces no noticeable symptoms in the early stage while causing complex complications in its advanced stage, making treatment difficult. Palliative therapy aims to relieve the symptoms of cancer patients and focuses on improving their quality of life. At present, five palliative therapies for advanced gastric carcinomas are offered: resection, gastrojejunostomy, stenting, chemotherapy, and radiotherapy. In recent years, palliative therapy has been used in the clinical treatment of advanced gastric carcinomas and related complications because of its efficacy in gastric outlet obstruction and gastric bleeding. In the future, multimodal and interdisciplinary palliative therapies can be applied to control general symptoms to improve patients’ condition, prolong their lifespan and improve their quality of life.

## 1 Introduction

Gastric carcinoma is a malignant tumor that starts from the epithelial tissue of the gastric mucosa. It is one of the most common malignant tumors in the world and the fifth leading cause of cancer-related death. Its mortality among tumors ranks fourth in the world and it has burdened economic development and global health care (1, 2). Early gastric carcinomas incur no obvious symptoms, and the most common clinical symptoms in the advanced stage are non-specific weight loss, persistent abdominal pain, dysphagia, hematemesis, anorexia, nausea, early satiety, and dyspepsia. Patients with advanced diseases in local areas or metastatic disease usually have obvious symptoms such as abdominal pain, potential ascites, weight loss, fatigue, and possible gastric outlet obstruction (3). The incidence of gastric carcinomas has obvious regional and gender differences. The morbidity rate of gastric carcinomas in men is twice that of women and the Asia-Pacific region has a high concentration of gastric carcinomas incidence in men, while Asia holds the highest incidence of this disease in women (4). It is estimated that by 2040, the number of new cancer cases worldwide will exceed 28.4 million, an increase of 47% compared with 2020. The increase is more significant in developing countries and the pressure of treating cancer will intensify (5, 6).

The current standard treatment for gastric carcinomas is mainly surgery, which delays disease progression and reduces tumor recurrence by directly resecting the patient’s tumor, lymph nodes, and damaged organs (7). Statistics show that the five-year survival rate for patients receiving radical resection for early gastric carcinomas is up to 90% (8), but recurrence and metastasis can still be observed in 20-60% of patients (9, 10). Since there are no noticeable symptoms early in the onset of gastric carcinomas, 80% of patients were in the advanced stage when they were diagnosed, and the prognosis was extremely poor because they missed the right time for treatment (11, 12). Complications such as intestinal obstruction, anemia, dumping syndrome, and gastrointestinal dysfunction may occur after surgical treatment. Although postoperative radiotherapy and chemotherapy can effectively prevent the recurrence of postoperative metastasis of gastric carcinomas, it often brings unbearable side effects, such as adverse reactions in the gastrointestinal tract and bone marrow suppression (13). The main goal of palliative therapy is to improve patients’ quality of life, and it is widely used in the treatment of advanced tumors as it relieves the symptoms of patients and solves their physical, psychological, and social problems, to improve the life of patients and their families (14). We focus on the application of palliative therapy in advanced gastric carcinomas, including the clinical application of these therapies for different advanced gastric carcinomas and related complications, with the aim to provide new plans for palliative therapy in the treatment of advanced gastric carcinomas and promote its application.

## 2 Significance of palliative therapy

The World Health Organization defines palliative care as a patient, family, or caregiver-centered medical service that focuses on the optimal management of painful symptoms (15). The long-term and repeated hospitalization, surgery, radiotherapy, multiple chemotherapy, and symptomatic treatment have seriously affected the psychological state of the patients. Palliative therapy is conducive to the smooth implementation of anti-cancer treatment and the best effect of treatment plans, especially for patients with gastric carcinomas chemotherapy (16). Relevant researchers believe that earlier active palliative therapy helps better control the general symptoms, prolong the survival of patients, and improve their living quality (17). At present, the main methods of palliative therapy for patients with gastric carcinomas include palliative resection (18), gastrojejunostomy (19), stent placement (20), chemotherapy (21) and radiotherapy (22).

## 3 Palliative gastrectomy

Palliative resection of gastric carcinomas refers to the surgery conducted to release complications and relieve symptoms for patients who cannot receive radical gastrectomy for various reasons, or for patients with potentially life-threatening complications, such as gastrointestinal bleeding, perforation, or obstruction (23). For patients with metastatic gastric carcinomas who have received first-line treatment, palliative resection can directly remove the obstruction, prevent the occurrence of critical complications such as gastric perforation and gastric bleeding, and effectively relieve symptoms such as obstruction, bleeding, pain and nausea, and might prolong the survival of patients when distant lymph node metastasis, liver metastasis, pelvic planting, peritoneal spread, invasion of surrounding organs occur (24, 25). A clinical study has found that palliative gastrectomy exerts a significant impact on the survival and short-term survival rate of patients with advanced gastric carcinomas (26). In addition, the surgical complications are controllable (27). Compared with patients who did not undergo palliative gastrectomy, the overall survival rate of those who received the palliative resection was significantly higher (28, 29). It was found (30) that among patients with locally advanced diseases without distant metastases, those who underwent palliative gastrectomy had a higher short-term survival rate. Relevant data of palliative total gastrectomy (PTG) for stage IV proximal gastric carcinomas suggested that compared with no operation, laparotomy and jejunostomy, the PTG of stage IV proximal gastric carcinomas better prolongs the survival time and improves the quality of life (31, 32). However, despite the feasibility and safety of PTG, it is necessary to select appropriate patients with stage IV proximal gastric carcinomas for this therapy and thoughtful preparations should be made during the perioperative period (33).

In addition, related studies suggest that age is closely related to the survival rate after palliative gastrectomy (34). Hsu et al. (35) found that in palliative gastrectomy, factors such as younger age (below 58 years old), better preoperative nutritional status, less lymph node involvement and postoperative chemotherapy had a significant impact on the improvement of postoperative survival rate. In the gastric carcinomas trial conducted by Dutch scholars (36), it was found that patients younger than 70 years old with only one metastatic site had longer overall survival after palliative resection and could benefit significantly from palliative gastrectomy. Some researchers also proposed the combination of palliative resection and chemotherapy. A recent clinical observation reported that a patient with advanced metastatic gastric cancer who received Nivolumab combined chemotherapy after palliative gastrectomy had no recurrence 32 months after surgery, and the patient achieved complete remission of symptoms and prolonged survival (37). Seo HS et al. (38) found that the 3-year survival rate of stage IV gastric cancer patients undergoing palliative gastrectomy after chemotherapy was higher than that of patients undergoing palliative gastrectomy alone (61.1 vs 16.2%, p = 0.003). These studies have shown that the combination of palliative resection and chemotherapy can improve the survival rate of patients compared with monotherapy (39).

Compared with gastric carcinomas patients without palliative resection, the general symptoms of patients after palliative gastrectomy were effectively relieved, and the short-term survival rate and postoperative survival time were improved (32). Although gastric carcinomas resection can alleviate the general symptoms of patients to a certain extent and improve the span and quality of their life, it is still necessary to pay attention to factors such as the age of the patient, the metastatic site of gastric carcinomas, lymphatic involvement, and patient’s preoperative nutrition because they also affect the prognosis of gastric carcinomas resection (35). In future studies, further clinical studies should be conducted on the related factors, and the post-resection chemotherapy and the combination of palliative resection and chemotherapy. Recent clinical trials of palliative gastrectomy on palliative treatment of advanced GC are shown in **Table 1**.

**Table 1:** Recent clinical trials of palliative gastrectomy on palliative treatment of advanced GC.

Author	Published	Interventions	Participants	Result	Conclusion
Kang YK (40)	2021	Palliative resection + S-1 Adjuvant Chemotherapy (SC))/palliative resection + Neoadjuvant Chemotherapy +Adjuvant chemotherapy (CSC)	266	CSC improved PFS versus SC (adjusted hazard ratio, 0.70; 95% CI, 0.52 to 0.95; stratified log-rank P = .023).	DOS chemotherapy is effective and tolerable in Korean patients with locally advanced gastric cancer.

## 4 Gastrojejunostomy

Gastrojejunostomy (GJ) is a standard palliative operation for gastric outlet obstruction (GOO), a common complication of gastric carcinomas, and is also the preferred treatment for malignant GOO in patients with advanced gastric carcinomas (41). Relevant clinical trials have demonstrated that the incidence of stent-related complications, re-obstruction, and re-intervention of gastrojejunostomy was low (42). Ouchi K et al. (43) investigated 95 patients to study the indicators of quality of life such as survival and hospital-free survival (HFS) of patients undergoing palliative gastric carcinomas surgery. The results showed that patients who received palliative therapies such as GJ and total gastrectomy had a moderate postoperative survival rate in the absence of peritoneal dissemination and adjacent peritoneal metastases. In improving the success rate of GJ, Prager LP et al. (44) conducted a study on the establishment of complete suture of GJ in machine-assisted Roux-en-Y gastric bypass and found that machine-assisted surgery can shorten the operation and improve suture, which is conducive to the recovery of the incision and the decrease of the risk of gastrojejunostomy stenosis as it reduces scar stenosis at the anastomotic point. In a retrospective study, investigators found that patients older than 80 years had a higher incidence of delayed gastric emptying and subsequent aspiration pneumonitis after GJ. Patients with gastric carcinomas induced mGO0 had an extremely poor prognosis, and the median survival time after gastrojejunostomy was less than 2 months (45), which suggests that gastrojejunostomy is not the best choice for elderly patients with gastric carcinomas.

Traditional GJ uses upper midline or subcostal incision, its cut is large and the safety and efficacy are low (46). With the advancement of laparoscopic techniques, recent studies have found that endoscopic ultrasound-guided gastrojejunostomy (EUS-GJ) had a smaller incision, fewer postoperative adverse events, and was safer and more effective (47–49). Boghossian M et al. (50) compared the efficacy of endoscopic ultrasound-guided gastroenterostomy (EUS-GE), duodenal stent (DS) placement, and surgical gastrojejunal (SGJ) therapies in the treatment of malignant GOO. It was found that compared with DS and SGJ, EUS-GE had a higher clinical success rate in the treatment of malignant GOO, lower incidence of serious adverse events and 30-day mortality, and shorter hospitalization. On W et al. (51) analyzed patients with gastric outlet obstruction who underwent continuous endoscopic ultrasound-guided gastroenterostomy (EUS-GJ) between August 2018 and March 2021, and found that the success rate of surgery was as high as 92%, the mean score of the gastric outlet obstruction scoring system was improved compared with SGJ (2.52 vs 0.68, p<0.01), and only 8% of patients reported adverse events, all of which were due to improper stent deployment and incurred gastric defect that requires endoscope to repair. This indicated that EUS-GJ could effectively improve the symptoms of patients with gastric outlet obstruction, and was safer and more efficient. When EUS-GJ is selected in future clinical practices, experienced endoscopists should be appointed to reduce the incidence of adverse events and the rate of reoperation.

The effect of GJ is not as obvious as that of total gastrectomy, but for patients with GOO, GJ can effectively improve their nutrition, avoid further obstruction, and reduce the occurrence of complications (52). Clinically, the overall condition of the patient should be comprehensively evaluated, the surgical standard should be defined, unnecessary morbidity should be avoided, cost-effective treatment should be achieved, and effective palliative therapy and suitable surgical methods should be selected (53). For patients with long life expectancy and good physical status, GJ is a preferred palliative therapy with a low rate of reintervention (54). It should be noted that the prognosis of GJ in elderly cancer patients is poor, and the survival time is short. The hospital should inform patients and their families of the risks, and ensure smooth communication between doctors and patients (55). In addition, with the advancement of science and technology, machine assistance such as endoscopes and ultrasound in surgery to improve precision, shorten the operation time, reduce the incidence of adverse events, relieve the pain, and promote the standardization of the operation (56). However, there are few relevant clinical studies at present, and palliative surgery research on this aspect can be increased in the future. Recent clinical trials of gastrojejunostomy on palliative treatment of advanced GC are shown in **Table 2**.

**Table 2:** Recent clinical trials of gastrojejunostomy on palliative treatment of advanced GC.

Author	Published	Interventions	Participants	Result	Conclusion
Lee HJ (57)	2019	laparoscopic distal gastrectomy (LDG)/D2 lymphadenectomy/open distal gastrectomy (ODG)	1050	Early morbidity rate was significantly lower after LDG (16.6%) than after ODG (24.1%; P = 0.003).Postoperative analgesics use and patients’ reported pain score were significantly lower after LDG.Postoperative hospital stay was shorter in LDG group (8.1 vs 9.3 d, P = 0.005).	Laparoscopic distal gastrectomy with D2 lymphadenectomy for locally advanced gastric cancer shows benefits in terms of lower complication rate, faster recovery, and less pain compared with open surgery.
Wei On (51)	2022	EUS-GJ	25	Both technical and clinical success were achieved in 92% (23/25) of patients. Long-term follow-up data were available for 21 of 23 patients and the re-intervention rate was 4.8% (1/21) over a median follow-up period of 162 (range 5-474) days.	EUS-GJ in carefully selected patients is an effective and safe procedure when performed by experienced endoscopists.

## 5 Endoscopic stent placement

In patients with in-progress and advanced gastric carcinomas, obstructions, such as gastric outlet obstruction and malignant intestinal obstruction are common complications (58). The main symptoms include pain, nausea, vomiting, abdominal distension, and decreased oral intake, which can lead to dehydration and malnutrition, undermining the quality of life of patients (59). ES is a palliative therapy for gastric carcinomas induced GOO with the significant advantage of a non-invasive approach (60). The stent can directly clear the digestive tract obstruction caused by gastric carcinomas minimize the pain of the patient and helps resume the patient’s normal diet (61) and improve their lives. Compared with GJ, ES has lower postoperative morbidity. However, secondary obstructions may reoccur due to tumor growth in ES, and the risk of re-operative intervention is higher than that of GJ, thus ES is preferred in elderly patients with advanced gastric carcinomas or multi-organ dysfunction (62, 63).

Self-expanding metal stent (SEMS) is the most common type of expandable stent characterized by softness and good expandability, and it is safe and effective for various gastrointestinal strictures (64). Jin et al. (65) investigated 207 patients who underwent metal stent implantation for gastric carcinomas induced GOO and it was found that the clinical success rate of stent implantation in all patients was 97%, the cumulative survival time reached 153 days, and the incidence of serious complications was only 1%, which indicated that metal stent implantation has clinical efficacy in the palliative therapy of GOO in patients with gastric carcinomas. In a study of the effect of endoscopic placement of SEMS or open gastric division gastrojejunostomy on the quality of life, researchers found that stenting can relieve the symptoms of pyloric obstruction in the short term and significantly improve the quality of life of patients (66). Krishnamoorthi et al. (67) compared the clinical success rate, GOO recurrence rate and safety indicators among duodenal SEMS, GJ and EUS-GE, and the results have shown that the clinical curative effect and clinical success rate of the three were similar. EUS-GE had a lower GOO recurrence rate (P=0.0036), and duodenal SEMS had a lower rate of procedure-related bleeding but a higher rate of reintervention (P=0.041). Other studies have also noted that SEMS placement is the optimal treatment for malignant GOO because it is less invasive. SEMS placement is more effective than palliative GJ in terms of earlier time to restoration of oral intake and a shorter hospital stay for patients with malignant gastric outlet obstruction (68, 69). The researchers compared the effects of GJ and SEMS as two palliative therapies for malignant GOO. They found that both methods were effective, but SEMS had a more significant short-term effect and thus it was more suitable for gastric carcinomas patients with short expected survival time (70). In terms of complication research, some researchers used the Ponsky-Gauderer technique to insert a ConfloTM PEG tube into the second part of the duodenum. The patient had an excellent tolerance of enteral nutrition and effective symptom relief and regained some weight without postoperative complications (71).

The palliative therapy of malignant outlet obstruction with stent implantation can significantly improve patients’ life, and this treatment has a high success rate especially for elderly patients with advanced gastric carcinomas and multiple organ dysfunction. Most of the implanted stents currently used in clinical use are metal stents. Metal stents are less invasive and thus can be further promoted. Endoscopic ultrasound-guided stent implantation is even safer, and it allows patients to diet after surgery and shortens their hospitalization. However, for obstruction caused by tissue or tumor growth, stent implantation has a higher reoperation rate and reintervention rate than gastrojejunostomy, therefore, it is more suitable for patients with shorter life expectancy. In addition, current studies suggest that few complications of stent implantation are identified and only limited relevant clinical research data have been collected, which means more samples should be involved in future research. Recent clinical trials of endoscopic stent placement on palliative treatment of advanced GC are shown in **Table 3**.

**Table 3:** Recent clinical trials of endoscopic stent placement on palliative treatment of advanced GC.

Author	Published	Interventions	Participants	Result	Conclusion
Park KB (72)	2001	Palliation +flexible covered metallic stents	24	The technical success rate was 75% (18 of 24 patients). Symptoms improved in 12 (67%) patients after the procedure.Twelve patients died during the follow-up period (mean survival, 4.3 months). The complication rate was 25% (six of 24 patients).	Flexible covered metallic stent placement can be useful for palliation in patients with malignant obstruction of the gastric outlet or duodenum.
Jeurnink SM (73)	2009	GJ / stent placement	39	Food intake improved more rapidly after stent placement than after GJJ (but long-term relief was better after GJJ, with more patients living more days with a GOO Scoring System score of 2 or more than after stent placement (72 vs 50 days, respectively; P = .05). There were also no differences in median survival (stent: 56 days vs GJJ: 78 days) and quality of life.	GJ was associated with better long-term results and is therefore the treatment of choice in patients with a life expectancy of 2 months or longer. Because stent placement was associated with better short-term outcomes, this treatment is preferable for patients expected to live less than 2 months.
Endo S (58)	2013	Self-expandable metallic stents	12	After stenting, a GOOSS score of 2 (soft solids only) or 3 (low-residue or full diet) was achieved in 13 (72%) patients. An improvement in the GOOSS score by one or more points was obtained in 16 (94%) patients. The median duration of fasting and hospital stay was 3 (range, 0-9) days and 18 (6-168) days, respectively. Chemotherapy was performed after stenting in 13 (72%) patients.	Gastroduodenal stents are thought to be feasible, safe, and effective for gastric outlet obstruction due to unresectable advanced gastric cancer, with rapid clinical relief and a short hospital stay.

## 6 Palliative chemotherapy

For patients with unresectable advanced gastric carcinomas, recurrent gastric carcinomas, and metastatic gastric carcinomas, systemic chemotherapy has become the standard treatment. Systemic chemotherapy is a kind of palliative therapy that aims to provide symptom relief and prolong survival (74). Treatment guidelines recommend a combination of palliative chemotherapy and chemotherapy drugs as first-line treatment because the therapy is non-invasive (75). For patients in the progression of gastric carcinomas, chemotherapy can reduce the postoperative recurrence rate (76). For patients with advanced gastric carcinomas, early palliative therapy combined with standard chemotherapy can deliver benefits to themselves, their families and society as the chemotherapy after surgery has a more significant impact on their survival (77). Currently, the most widely accepted standard palliative chemotherapy regimen worldwide is the combination of 5-fluorouracil (5-FU) and platinum analogs, in which 5-FU can be replaced by S-1 or capecitabine, and cisplatin can be replaced by oxaliplatin (74). Countries in East Asia typically use standard chemotherapy regimens of S-1 or capecitabine & oxaliplatin based on the ACTS-GC (78) or CLASSIC (79) trials for palliative chemotherapy.

Research data have proved that palliative chemotherapy for advanced gastric carcinomas can improve the survival rate of patients, reduce the burden of locally advanced tumors, and relieve common clinical symptoms such as pain, dysphagia, and malignant obstruction (80). Results of a multicenter study in Japan showed that patients with stage Il or Ill gastric carcinomas who underwent gastrectomy with extended (D2) lymph node dissection had 10% higher overall survival after adjuvant S-1 therapy than those who just underwent surgery (80.1% VS 70.1%) (78, which indicates that S-1 is an effective adjuvant therapy for patients with D2 resection of locally advanced gastric carcinomas in East Asia (81).

In addition to standardized palliative chemotherapy regimens, new palliative chemotherapy regimens are also explored. Japan Clinical Oncology Group (JCOG) conducted a multicenter phase II trial to evaluate the safety and efficacy of preoperative chemotherapy S-1&cisplatin combined with gastrectomy in plastic gastritis (type 4) or large ulcero-invasive-type (type 3) gastric carcinomas patients. The results showed that the treatment completion rate of this therapy reached 73.5%, higher than the threshold of 45%, and treatment-related death (TRD) was 2%, confirming that the therapy was safe and effective (81). Zhu YJ et al. (82) found that acupuncture therapy helped relieve symptom burden and reduce toxic and side effects in patients receiving palliative chemotherapy.

Peritoneal metastasis refers to the formation of peritoneal tumors in the abdominal cavity, and GC patients with peritoneal metastasis generally have a poor prognosis (83). In the past few decades, many techniques have been applied for the treatment of GC patients with peritoneal metastasis, such as repeated intraperitoneal chemotherapy combined with systemic chemotherapy, and pressurized intraperitoneal aerosol chemotherapy (PIPAC), with considerable clinical efficacy (84). Several phase II trials and one phase III trial conducted by Kobayashi et al. (85) confirmed that repeated intraperitoneal chemotherapy based on taxanes combined with systemic chemotherapy had little toxicity in the treatment of gastric carcinomas with peritoneal metastases, and the 1-year overall survival rate of patients ranged 67% to 91%, suggesting the safety and feasibility. Recent studies also found that pressurized intraperitoneal aerosol chemotherapy was a safe and feasible treatment with low in-hospital morbidity and mortality, and it could effectively improve the quality of life of patients with peritoneal metastases (86). Alyami et al. (87) performed a retrospective analysis on patients with gastric carcinomas peritoneal metastasis (PM) treated with PIPAC. The results indicated that the overall complication rate and major complication rate (CTCAE-III, IV) was 6.1% and 3.1%, and 14.3% of the patients could receive tumor resection during the treatment, which shows that PIPAC is a safe and effective drug delivery system for the treatment of PM in gastric carcinomas. Based on the retrospective analysis, it was found that for the majority of GC patients with unresectable PM, PIPAC was a well-tolerated option providing good quality of life stability in terms of symptom relief (88). These studies provide new ideas for the chemotherapy of gastric carcinoma peritoneal metastasis. In future research, we can further strengthen the exploration of new technologies to identify their potential and possibilities. For example, these chemotherapy methods can be used as a “neoadjuvant” strategy to qualify those unfit GC patients for gastric carcinomas resection.

The effectiveness of palliative chemotherapy in elderly patients with gastric carcinomas has been receiving much attention. Available data suggest that combination chemotherapy can be an option for elderly patients with metastatic gastric carcinomas classified as not frail according to Comprehensive Geriatric Assessment (CGA) (89). ShengLi He et al. studied the use of capecitabine “rhythmic” chemotherapy in elderly patients with gastric carcinomas and they found that the disease control rate (DCR) of chemotherapy exceeded 50% (95% CI 25.7–67.8) at 8 weeks, and there was no report of IV hematological toxicity or febrile neutropenia, suggesting that palliative chemotherapy is safe and effective in patients with advanced gastric carcinomas (90). Liao et al. divided 306 patients receiving palliative chemotherapy into two groups at the age of 70 years and compared the clinical demographics, prognosis and hematological toxicity of chemotherapy between the two groups. It was found that the progression-free survival and overall survival were the same and the incidence of hematological toxicity was similar (91), which suggests that age is not a limiting condition for palliative chemotherapy in elderly patients with metastatic gastric carcinomas.

Palliative chemotherapy can improve the patient’s symptoms and quality of life, and is considered a safe and promising treatment for gastric carcinomas (92). In addition to studying existing palliative chemotherapy regimens, research should also be done on the role of new palliative regimens that combine palliative chemotherapy with other treatments for patients with advanced gastric carcinomas (93). The condition of patients with advanced gastric carcinomas is complex and they are often complicated with peritoneal metastasis, thus age and physical condition should be considered in the selection of palliative chemotherapy. Research on the selection of intraperitoneal chemotherapy regimens, follow-up treatment after failure of initial treatment, conversion surgery after control of peritoneal metastases and its survival effect are remains insufficient,and there is still room for improvement (94). PIPAC may be a feasible option for relieving symptoms of unresectable PM but more random test research is needed. For elderly and frail patients, their overall condition should be accessed and lower-intensity palliative chemotherapy can be used. Further prospective, randomized, controlled trials can be conducted in the future to verify the results. Recent clinical trials of chemotherapy on palliative treatment of advanced gastric cancer are shown in **Table 4**.

**Table 4:** Recent clinical trials of chemotherapy on palliative treatment of advanced gastric cancer.

Author	Published	Interventions	Participants	Result	Conclusion
Shchepotin IB (95)	1999	systemic intravenous chemotherapy/superselective intra-arterial chemotherapy	74	The median survival time in the control group and after intravenous chemotherapy was only 91 and 96 days, respectively, as compared to 401 days in the patients receiving intra-arterial chemotherapy.	The results confirmed that superselective intra-arterial chemotherapy conferred a highly significant survival advantage compared to control or systemic intravenous chemotherapy adjusted for all patient characteristics (P <0.0001).
Bang YJ (96)	2010	chemotherapy+Trastuzumab/ chemotherapy	584	The most common adverse events in both groups were nausea (trastuzumab plus chemotherapy, 197 (67%) vs chemotherapy alone, 184 (63%)), vomiting (147 (50%) vs 134 (46%)), and neutropenia (157 (53%) vs 165 (57%)).	Trastuzumab in combination with chemotherapy can be considered as a new standard option for patients with HER2-positive advanced gastric or gastro-oesophageal junction cancer.
Li XD (97)	2011	fluorouracil + cisplatin (PCF)/ oxaliplatin + fluorouracil + leucovorin (FOLFOX-4)	94	No significant difference was observed in overall response rate (ORR),disease control rate (DCR), median survival times (MSTs) or 1-year survival rate between the two groups (48.0% and 45.5%、82.0% and 81.8%、 10.8 and 9.9 mo、36.0% and 34.1%,respectively).	Patients with AGC have a similar response rate to PCF and FOLFOX-4 regimens with a similar survival rate. The PCF and FOLFOX-4 regimens are efficacious and tolerable as a promising therapy for AGC.
Ajani JA (98)	2013	S-1/cisplatin (CS) + infusional 5-FU/cisplatin (CF)	1053	Overall survival (OS) in CS (8.6 months) was statistically noninferior to CF (7.9 months) (hazard ratio (HR) = 0.92 (two-sided 95% confidence interval (CI), 0.80-1.05)) for any margin equal to or greater than 1.05. Statistically significant safety advantages for the CS arm were observed.Treatment-related deaths were significantly lower in the CS arm than the CF arm (2.5% and 4.9%, respectively; P<0.047).	CS is noninferior to CF with a better safety profile and provides a new treatment option for patients with advanced gastric carcinoma.
Dong L (99)	2014	S-1 + irinotecan + oxaliplatin (TIROX) /docetaxel + cisplatin + flurouracil (DCF)	60	The response rate (complete response + partial response) was significantly higher in the TIROX group (18/30 patients; 60.0%) compared with the DCF group (10/30 patients; 33.3%). The rates of grade III-IV leucopenia and neurotoxicity were significantly higher in the TIROX group than the DCF group.	The TIROX regimen was effective for the treatment of advanced gastric cancer, but it was associated with leucopenia and neurotoxicity.
Ochenduszko S (100)	2015	epirubicin + oxaliplatin + capecitabine (EOX) / docetaxel + cisplatin + 5FU + leucovorin (mDCF)	56	progression-free survival was 6.4 and 6.8 months, respectively (p = 0.440). Two-year survival rate was 22.2 % with mDCF compared to 5.2 % with EOX.There was no statistically significant difference in the rates of grade 3-4 adverse events (EOX 79.3 vs. mDCF 61.5 %; p = 0.234).	The mDCF regimen was associated with a statistically nonsignificant 2.4-month longer median overall survival without an increase in toxicity.
Quintero-Aldana G (101)	2015	docetaxel + cisplatin	55	The objective response rate was 42.3 % (95 % CI 28.9-55.7), the median time to progression was 5.5 months (95 % CI 4.0-7.0), and the median overall survival was 8.9 months (95 % CI 6.0-11.9)	Biweekly administration of docetaxel and cisplatin in advanced gastric cancer has a manageable toxicity profile and shows a promising antitumour activity as a first-line therapy.
Yamada Y (102)	2015	S-1 /cisplatin + S-1(CS)	685	In per-protocol population, SOX (n = 318) was noninferior to CS(n = 324) in PFS (median, 5.5 versus 5.4 months.The median OS for SOX and CS were 14.1 and 13.1 months, respectively. In the intention-to-treat population,the HRs in PFS and OS were 0.979 (95% CI 0.821-1.167) and 0.934 (95% CI 0.786-1.108), respectively.	SOX is as effective as CS for AGC with favorable safety profile, therefore SOX can replace CS.
Chen X (103)	2016	S-l + cisplatin + concurrent chemoradiotherapy (SCCC) / cisplatin + concurrent chemoradiotherapy (CCC)	144	The median overall survival durations were 11.7 monthsand 9.5 months in SCCC and CCC groups, respectively (P = 0.041). The median progression-free survival durations were 10.6 months for SCCC and 8.8 months for CCC (P = 0.046). The toxicity profile was similar in both groups.	SCCC showed more promising safety and efficacy than CCC in Chinese patients with AGC. In addition, the toxicities were also acceptable in both groups.
Wang J (104)	2016	docetaxel + cisplatin + and fluorouracil/ modified DCF (mDCF)	243	Compared with DCF, the mDCF regimen significantly improved PFS and OS and ORR.Grade 3 and grade 4 treatment-related adverse events occurred in 77.3 % of patients who received the mDCF regimen versus 46.1% of patients who received CF (P < 0.001).	The mDCF regimen, compared with CF, achieved efficacy comparable but significantly prolonged PFS and OS and enhanced ORR of Chinese patients with advanced gastric cancer,besides that with fewer toxicities, which is appropriate for the Chinese population.
Fushida S (105)	2016	Weekly paclitaxel (wPTX) / Weekly paclitaxel + valproic acid (wPTX + VPA)	66	The median OS was 9.8 months in the wPTX group and 8.7 months in the wPTX plus VPA group (hazard ratio 1.19; 95% CI 0.702-2.026; P=0.51). The median progression-free survival was 4.5 months in the wPTX group and 3.0 months in the wPTX plus VPA group (hazard ratio 1.29; 95% CI 0.753-2.211; P=0.35). Grade 3-4 adverse events were neutropenia (3.1%), pneumonia (1.6%), liver injury (1.6%), brain infarction (1.6%), and rupture of aorta (1.6%).	No statistically significant difference was observed between wPTX and wPTX plus VPA for OS.
Sah BK (106)	2020	FLOT Chemotherpy Regimen (5-FU+CF+Docetaxel+Oxaliplatin)/SOX Chemotherapy Regimen (Oxaliplatin+TGO)	74	There are no significant differences in adverse effects or postoperative morbidity and mortality between the two groups. No significant differences in the proportion of tumor regression grading between the FLOT group and the SOX group are found. Complete or subtotal TRG is 20.0% in the FLOT group versus 32.4% in the SOX group.	TFor locally advanced gastric cancer, there was no significant difference in efficacy between neoadjuvant FLOT and SOX regimens.
Kawakami H (107)	2020	S-1 + cisplatin (SP) / capecitabine + cisplatin (XP)	101	SP (n = 44-50) showed a longer progression-free survival (6.4 versus 5.1 months; hazard ratio (HR), 0.666; P = 0.062), overall survival (14.8 versus 10.6 months; HR, 0.695; P = 0.099), and time to treatment failure (4.6 versus 3.6 months; HR, 0.668; P = 0.045) as well as a higher disease control rate (86.4% versus 68.1%, P = 0.149) compared with XP (n = 47-51).	A significant survival advantage for SP over XP was apparent in patients with a performance status of 0, a differentiated-type tumor histology, or a primary tumor localization to the upper portion of the stomach.SP in the first-line setting for patients with HER2-negative advanced or recurrent GC with a recurrence-free interval of ≥ 6 months.
Zhu XD (108)	2022	oxaliplatin + capecitabine(XELOX) / epirubicin + oxaliplatin + capecitabine (EOX)	448	The median PFS (mPFS) was 5.0 months (95% confidence interval (CI) = 4.5-6.0 months) in the XELOX arm and 5.5 months (95% CI = 5.0-6.0 months) in the EOX arm (hazard ratio (HR) = 0.989, 95% CI = 0.812-1.203; Pnon-inferiority = 0.003).	As the triplet regimen and had a better safety profile and QoL as a first-line treatment for AGC patients.
Bin Y (109)	2022	docetaxel+ oxaliplatin +S-1	78	The median OS in the group of D-SOX group (intraperitoneal infusion of docetaxel) was significantly higher than that in the aDOS group (intravenous docetaxel).The ascites control rate in the D-SOX group was 58.9% and 30.8% in DOS group and patients in the D-SOX group had lower grade 3/4 blood toxicity than that in the DOS group (26% vs 54%, P = 0.01).	Compared with traditional systemic chemotherapy, docetaxel intraperitoneal infusion combined with chemotherapy has better therapeutic effect on GC ascites, with better survival benefit and tolerance and less hematological toxicity, which is worthy of further research and clinical application.
Rosati G (110)	2022	fractionated docetaxel + oxaliplatin + capecitabine (low-tox)/epirubicin + oxaliplatin +capecitabine (eox)	169	Median PFS was 6.3 months (95% confidence interval (CI) 5.0-8.1) in EOX vs 6.3 months (95% CI 5.0-7.8) in low-TOX, without statistical difference. OS was comparable in the two arms: 12.4 in EOX(95% CI 9.1-19.2) vs 11.5 months in low-TOX (95% CI 8.6-15.0). ORR was 33% and 24%, while DCR was 68% and 67%, respectively.	A triplet regimen based on the fractional dose of docetaxel achieves no improvement over EOX which remains a potential standard treatment in many patients with inoperable, locally advanced or metastatic GC.

## 7 Palliative radiotherapy

Palliative radiotherapy refers to the application of radiotherapy to the primary tumor and metastases of advanced malignant tumors to relieve pain, improve symptoms, and prolong life (111). Palliative radiotherapy for advanced GC patients is safe and effective. It is well-tolerated by patients and can greatly improve the quality of life of patients with poor prognoses (112).

RT has high efficacy for local gastric carcinomas, and its total effective rate of treating bleeding, pain and obstructive symptoms can reach 74%, 67% and 68% respectively. RT works for elderly patients who are not suitable for chemotherapy, patients with poor performance status, or patients receiving chemotherapy (113). RT has the most obvious curative effect in the treatment of gastric bleeding (114). Studies have found that for patients with unresectable advanced gastric carcinomas, RT is minimally invasive which can achieve hemostasis in a short time and effectively treat gastric carcinomas with gastric bleeding and obstruction (115). In addition, multi-center studies have confirmed that palliative radiotherapy had considerably improved mildly toxic anemia (116).

Although radiation therapy has a certain effect on gastric hemostasis, the mechanism is not clear, and the radiation dose needs to be further determined (117). The researchers used low-dose 6Gy to treat gastric bleeding, and the results suggest that LSP-RT (low-dose, short-course palliative RT) for the treatment of GC bleeding is safe, effective, and reproducible in the case of rebleeding. This treatment may help improve the quality of life of patients (118). Asakura et al. used RT at a dose of 30 Gy to moderate bleeding in advanced gastric carcinomas, achieving hemostasis in 73% of patients. Considering the median actuarial time to rebleeding (3.3 months), this tiered dose regimen appears to be adequate, especially for patients with poor prognoses (119).

Hemorrhage and obstruction are common complications in patients with advanced gastric carcinomas. In clinical practice, RT is used to treat bleeding and obstruction in advanced gastric carcinomas (120). However, the number of clinical observations on the relationship between radiation dose and rebleeding is relatively small, and more randomized controlled trials are needed TO determine the optimal dosage and distribution scheme. Recent clinical trials of radiotherapy on palliative treatment of advanced gastric cancer are shown in **Table 5**.

**Table 5:** Recent clinical trials of radiotherapy on palliative treatment of advanced gastric cancer.

Author	Published	Interventions	Participants	Result	Conclusion
Shao ZY (121)	2008	oxaliplatin + 5-fluorouracil/leucovorin calcium + concurrent radiotherapy (I)/only chemotherapy(II)	83	Patients treated with radiotherapy combined with concurrent chemotherapy had a higher R0 resection rate(77.8% vs 57.1%, P = 0.161), The mean survival times of the resectable patients were 45 months and 28 months respectively, and the 2-year overall survival (OS) of the resectable patients were 65.8% and 56.3% respectively (P = 0.371). The mean survival times of the un-resectable patients were 14 months and 9 months respectively, and the 2-year OS rates of the un-resectable patients were 28.3% and 20.7% respectively (P = 0.017)	Radiotherapy combined with concurrent chemotherapy may be an effective and well-tolerated regimen in patients with advanced and metastatic gastric cancer.
Yoshikawa T (122)	2009	paclitaxel + cisplatin +45 Gy	9	A total of nine patients were enrolled, of which six were into level 1 (PTX 60 mg/m(2) and CDDP 20 mg/m(2)) and three into level -1 (PTX 50 mg/m(2) and CDDP 20 mg/m(2)). At level 1, one patient developed grade 3 fatigue, and the other experienced grade 5 DIC, grade 5 pneumonia, grade 4 thrombocytopenia, grade 3 hyponatremia, and grade 3 esophagitis as dose-limiting toxicities. A palliative effect was observed in eight of nine patients; six of six patients at level 1 and two of three at level -1.	PTX 50 mg/m(2) and CDDP 20 mg/m(2) given biweekly with concurrent radiation therapy of 45 Gy were well tolerated.
Kwon HC (123)	2010	chemoradiation + 5-fluorouracil/cisplatin (FP) (A) or FP (B)	61	The median follow-up duration was 77.2 months (range 24-92.8 months). We did not find any difference in 3-year disease-free survival between arm A and B (80.0 vs 75.2%, respectively; P = 0.887). There was no significant difference between the arms in 5-year disease-free survival (76.7 vs 59.1%, respectively; P = 0.222) or overall survival (70.1 vs 70.0%, respectively; P = 0.814).	There is no any conclusion about the benefit of adding radiation to adjuvant chemotherapy.
An JY (124)	2013	chemoradiotherapy (CCRTx)/chemotherapy (CTx)	74	Eleven patients (14.8%) demonstrated pathologic response favorable and the remaining 63 patients (85.2%) were response others. Chemoradiotherapy (CCRTx) rather than chemotherapy (CTx) was the only predictive factor for a favorable pathologic response.	Favorable pathologic response has been achieved after neoadjuvant treatment, CCRTx appears to be more effective for achieving good pathologic response than CTx
Boda-Heggemann J (125)	2013	adjuvant intensity-modulated radiation (IMRT)/capecitabine +oxaliplatin radiochemotherapy(XELOX)/three-dimensional conformal radiation therapy(3D-CRT)	65	Median overall survival times (OS) were 18 and 43 months in the 3D-CRT and IMRT groups, respectively (p = 0.0602). Actuarial 5-year OS rates were 26 and 47%, respectively. Within the IMRT group, earlier cohort and XELOX gave better results than 5-FU/FA in terms of OS, but this difference was not statistically significant. Median disease-free survival(DFS) times were 14 and 35 months in the 3D-CRT and IMRT groups, respectively (p = 0.0693). Actuarial 5-year DFS rates were 22 and 44%, respectively. No late toxicity exceeding grade 3 or secondary tumors were observed.	After a median follow-up period of over 5 years, OS and DFS were improved in the IMRT/XELOX treated patients compared to the 3D-CRT/5-FU/FA group. Long-term observation revealed no clinical indications of therapy-induced secondary tumors or renal toxicity.
Nishikawa K (126)	2018	capecitabine + cisplatin (XP)/S-1 + cisplatin (SP)	110	In 110 eligible patients, 24-week PFS was higher in both groups (SP 50.9%, XP 43.5%) than the protocol-specified threshold of 40%. The median PFS for SP versus XP was 5.6 and 5.1 months (hazard ratio (HR), 1.126; p = 0.5626); OS was 13.5 and 12.6 months (HR, 0.942; p = 0.7769) and the ORR was 42.4% and 69.4% (p = 0.0237), respectively.	XP and SP are comparable and can be recommended as control arms in a phase III study for AGC.
Wang F (127)	2021	neoadjuvant chemoradiotherapy (NACRT) + postoperative adjuvant XELOX (Oxaliplatin +Capecitabine) chemotherapy/postoperative adjuvant chemotherapy (ACT) with XELOX	60	Patients in NACRT group showed a significantly higher R0 resection rate (84.6% vs 56.7%, p = 0.029),lower loco-regional recurrence rate (36.7% vs 11.5%, p = 0.039), longer PFS (p = 0.019) and freedom from locoregional progression(FFLP) (p = 0.004) than patients in ACT group, while there was no difference in OS (p = 0.215) and in toxicity incidence (p > 0.05).	NACRT combined with postoperative adjuvant XELOX chemotherapy can improve R0 resection rate, reduce loco-regional recurrence, prolong PFS and FFLP without increasing the incidence of postoperative complications in patients with LAGC.

## 8 Conclusions and directions for future development

The occurrence of GC is occult, and the best way to improve advanced gastric carcinomas is prevention based on early screening. When gastric carcinomas progress to advanced stage, early and active palliative therapy as well as multi-modal and interdisciplinary methods should be applied to control general symptoms to improve patients’ condition, their survival and their quality of life. When palliative resection is used to treat advanced gastric carcinomas, attention should be paid to the age of the patient, the metastatic site and the number of cancers. If the tumor is limited to only one metastatic site and the patient is younger than 70 years old, patients can have a good prognosis with resection. Stenting can quickly relieve the obstruction of patients and improve their quality of life in a short period, but the complications of stenting and the risk of reoperation should be noted. Further well-designed randomized clinical studies can be conducted to compare different techniques related to palliative surgery, and different palliative therapy methods can also be used in combination to improve the treatment effect of palliative therapy.

## Author contributions

AH and Y-ZP were responsible for the study concept and design, as co-corresponding author. X-FL and Y-HL drafted the manuscript together, equal contribution, listed as co-first author, and all authors revised it for important intellectual content. The work reported in the paper has been performed by the authors, unless clearly specified in the text. All authors contributed to the article and approved the submitted version.

## References

[1] Johnston FM Beckman M. Updates on management of gastric cancer. Curr Oncol Rep (2019) 21(8):67. doi: 10.1007/s11912-019-0820-4 31236716

[2] Machlowska J Baj J Sitarz M Maciejewski R Sitarz R. Gastric cancer: Epidemiology, risk factors, classification, genomic characteristics and treatment strategies. Int J Mol Sci (2020) 21(11):4012. doi: 10.3390/ijms21114012 32512697 PMC7312039

[3] Dreznik A Purim O Idelevich E Kundel Y Sulkes J Sulkes A. Gastric cancer: biology and clinical manifestations in Israel. J Surg Oncol (2012) 105(3):316–22. doi: 10.1002/jso.22078 21882200

[4] Sung H Ferlay J Siegel RL Laversanne M Soerjomataram I Jemal A. Global cancer statistics 2020: GLOBOCAN estimates of incidence and mortality worldwide for 36 cancers in 185 countries. CA Cancer J Clin (2021) 71(3):209–49. doi: 10.3322/caac.21660 33538338

[5] Rahib L Wehner MR Matrisian LM Nead KT. Estimated projection of US cancer incidence and death to 2040. JAMA Netw Open (2021) 4(4):e214708. doi: 10.1001/jamanetworkopen.2021.4708 33825840 PMC8027914

[6] Bray F Parkin DM African Cancer Registry Network. Cancer in sub–Saharan Africa in 2020: A review of current estimates of the national burden, data gaps, and future needs. Lancet Oncol (2022) 23(6):719–28. doi: 10.1016/S1470-2045(22)00270-4 35550275

[7] Yura M Yoshikawa T Otsuki S Yamagata Y Morita S Katai H. The therapeutic survival benefit of splenic hilar nodal dissection for advanced proximal gastric cancer invading the greater curvature. Ann Surg Oncol (2019) 26(3):829–35. doi: 10.1245/s10434-018-07122-9 30569298

[8] Smyth EC Nilsson M Grabsch HI van Grieken NC Lordick F. Gastric cancer. Lancet (2020) 396(10251):635–48. doi: 10.1016/S0140-6736(20)31288-5 32861308

[9] Isobe Y Nashimoto A Akazawa K Oda I Hayashi K Miyashiro I. Gastric cancer treatment in Japan: 2008 annual report of the JGCA nationwide registry. Gastric Cancer (2011) 14(4):301–16. doi: 10.1007/s10120-011-0085-6 PMC3196643 21894577

[10] Rivera F Vega–Villegas ME López–Brea MF. Chemotherapy of advanced gastric cancer. Cancer Treat Rev (2007) 33(4):315–24. doi: 10.1016/j.ctrv.2007.01.004 17376598

[11] Casamayor M Morlock R Maeda H Ajani J. Targeted literature review of the global burden of gastric cancer. Ecancermedicalscience (2018) 12:883. doi: 10.3332/ecancer.2018.883 30679950 PMC6345079

[12] Zeng H Chen W Zheng R Zhang S Ji JS Zou X. Changing cancer survival in China during 2003–15: A pooled analysis of 17 population–based cancer registries. Lancet Glob Health (2018) 6(5):e555–67. doi: 10.1016/S2214-109X(18)30127-X 29653628

[13] Tanaka Y Fujii S Kusaka T Kokuryu H. Gastric mucosal carcinoma with pyloric stenosis. Intern Med (2021) 60(5):807–8. doi: 10.2169/internalmedicine.5924-20 PMC7990621 33028778

[14] Radbruch L De Lima L Knaul F Wenk R Ali Z Bhatnaghar S. Redefining palliative care–a new consensus–based definition. J Pain Symptom Manage (2020) 60(4):754–64. doi: 10.1016/j.jpainsymman.2020.04.027 PMC8096724 32387576

[15] Piñeros M Znaor A Mery L Bray F. A global cancer surveillance framework within noncommunicable disease surveillance: Making the case for population–based cancer registries. Epidemiol Rev (2017) 39(1):161–9. doi: 10.1093/epirev/mxx003 28472440

[16] Deo SVS Kumar N Rajendra VKJ Kumar S Bhoriwal SK Ray M. Palliative surgery for advanced cancer: Clinical profile, spectrum of surgery and outcomes from a tertiary care cancer centre in low–Middle–Income country. Indian J Palliat Care (2021) 27(2):281–5. doi: 10.25259/IJPC_399_20 PMC8428898 34511797

[17] Harada K Zhao M Shanbhag N Baba H Ajani JA. Palliative care for advanced gastric cancer. Expert Rev Anticancer Ther (2020) 20(7):575–80. doi: 10.1080/14737140.2020.1781620 PMC7415645 32543938

[18] Samarasam I Chandran BS Sitaram V Perakath B Nair A Mathew G. Palliative gastrectomy in advanced gastric cancer: is it worthwhile? ANZ J Surg (2006) 76(1–2):60–3. doi: 10.1111/j.1445-2197.2006.03649.x 16483298

[19] Del Piano M Ballarè M Montino F Todesco A Orsello M Magnani C. Endoscopy or surgery for malignant GI outlet obstruction? Gastrointest Endosc (2005) 61(3):421–6. doi: 10.1016/s0016-5107(04)02757-9 15758914

[20] Espinel J Sanz O Vivas S Jorquera F Muñoz F Olcoz J. Malignant gastrointestinal obstruction: endoscopic stenting versus surgical palliation. Surg Endosc (2006) 20(7):1083–7. doi: 10.1007/s00464-005-0354-8 16703436

[21] Cunningham SC Schulick RD. Palliative management of gastric cancer. Surg Oncol (2007) 16(4):267–75. doi: 10.1016/j.suronc.2007.08.004 17881220

[22] Izuishi K Mori H. Recent strategies for treating stage IV gastric cancer: Roles of palliative gastrectomy, chemotherapy, and radiotherapy. J Gastrointestin Liver Dis (2016) 25(1):87–94. doi: 10.15403/jgld.2014.1121.251.rv2 27014758

[23] Peng W Ma T Xu H Wu Z Wu C Sun G. Survival benefits of palliative gastrectomy in stage IV gastric cancer: A propensity score matched analysis. J Gastrointest Oncol (2020) 11(2):376–85. doi: 10.21037/jgo.2020.01.07 PMC7212110 32399278

[24] Sun J Song Y Wang Z Chen X Gao P Xu Y. Clinical significance of palliative gastrectomy on the survival of patients with incurable advanced gastric cancer: A systematic review and meta–analysis. BMC Cancer (2013) 13:577. doi: 10.1186/1471-2407-13-577 24304886 PMC4235220

[25] Park JY Yu B Park KB Kwon OK Lee SS Chung HY. Impact of palliative gastrectomy in patients with incurable gastric cancer. Med (Kaunas) (2021) 57(3):198. doi: 10.3390/medicina57030198 PMC7996496 33652574

[26] Kim DY Joo JK Park YK Ryu SY Kim YJ Kim SK. Is palliative resection necessary for gastric carcinoma patients? Langenbecks Arch Surg (2008) 393(1):31–5. doi: 10.1007/s00423-007-0206-1 17593384

[27] Chen XJ Chen GM Wei YC Yu H Wang XC Zhao ZK. Palliative gastrectomy versus gastrojejunostomy for advanced gastric cancer with outlet obstruction: A propensity score matching analysis. BMC Cancer (2021) 21(1):188. doi: 10.1186/s12885-021-07904-7 33622258 PMC7903659

[28] Dittmar Y Voigt R Heise M Rabsch A Jandt K Settmacher U. Indikationen und ergebnisse der palliativen magenresektion beim fortgeschrittenen magenkarzinom (Indications and results of palliative gastric resection in advanced gastric carcinoma). Zentralbl Chir (2009) 134(1):77–82. doi: 10.1055/s-0028-1098793 19242887

[29] Cowling J Gorman B Riaz A Bundred JR Kamarajah SK Evans RPT. Peri–operative outcomes and survival following palliative gastrectomy for gastric cancer: A systematic review and meta–analysis. J Gastrointest Cancer (2021) 52(1):41–56. doi: 10.1007/s12029-020-00519-4 32959118 PMC7900337

[30] Amaral L Mendes J Martins P Bernardo L Quintanilha R Santos V. Survival benefits of palliative gastric cancer resection – a regional center experience. Hepatogastroenterology (2012) 59(117):1651–6. doi: 10.5754/hge10201 22683985

[31] Lupaşcu C Andronic D Ursulescu C Vasiluţă C Raileanu G Georgescu ST. Palliative gastrectomy in patients with stage IV gastric cancer–our recent experience. Chirurgia (Bucur) (2010) 105(4):473–6. 20941968

[32] Wu P Wang P Ma B Yin S Tan Y Hou W. Palliative gastrectomy plus chemotherapy versus chemotherapy alone for incurable advanced gastric cancer: A meta–analysis. Cancer Manag Res (2018) 10:4759–71. doi: 10.2147/CMAR.S179368 PMC6208494 30464590

[33] Zhang JZ Lu HS Huang CM Wu XY Wang C Guan GX. Outcome of palliative total gastrectomy for stage IV proximal gastric cancer. Am J Surg (2011) 202(1):91–6. doi: 10.1016/j.amjsurg.2010.05.014 21600557

[34] Huang KH Wu CW Fang WL Chen JH Lo SS Wang RF. Palliative resection in noncurative gastric cancer patients. World J Surg (2010) 34(5):1015–21. doi: 10.1007/s00268-010-0467-7 20145923

[35] Hsu JT Liao JA Chuang HC Chen TD Chen TH Kuo CJ. Palliative gastrectomy is beneficial in selected cases of metastatic gastric cancer. BMC Palliat Care (2017) 16(1):19. doi: 10.1186/s12904-017-0192-1 28288593 PMC5348866

[36] Hartgrink HH Putter H Klein Kranenbarg E Bonenkamp JJ van de Velde CJ Dutch Gastric Cancer Group. Value of palliative resection in gastric cancer. Br J Surg (2002) 89(11):1438–43. doi: 10.1046/j.1365-2168.2002.02220.x 12390389

[37] Dai P Rao X Zhang X Qiu E Wu G Lin Y. Case report: Complete remission of a patient with metastatic gastric cancer treated with nivolumab combined with chemotherapy after palliative surgery. Front Immunol (2022) 13:908558. doi: 10.3389/fimmu.2022.908558 35844567 PMC9278084

[38] Seo HS Song KY Jung YJ Park SM Jeon HM Kim W. Radical gastrectomy after chemotherapy may prolong survival in stage IV gastric cancer: A Korean multi–institutional analysis. World J Surg (2018) 42(10):3286–93. doi: 10.1007/s00268-018-4635-5 29717344

[39] Tokunaga M Makuuchi R Miki Y Tanizawa Y Bando E Kawamura T. Surgical and survival outcome following truly palliative gastrectomy in patients with incurable gastric cancer. World J Surg (2016) 40(5):1172–7. doi: 10.1007/s00268-015-3398-5 26754075

[40] Kang YK Yook JH Park YK Lee JS Kim YW Kim JY. PRODIGY: A phase III study of neoadjuvant docetaxel, oxaliplatin, and s–1 plus surgery and adjuvant s–1 versus surgery and adjuvant s–1 for resectable advanced gastric cancer. J Clin Oncol (2021) 39(26):2903–13. doi: 10.1200/JCO.20.02914 PMC8425847 34133211

[41] Min SH Son SY Jung DH Lee CM Ahn SH Park DJ. Laparoscopic gastrojejunostomy versus duodenal stenting in unresectable gastric cancer with gastric outlet obstruction. Ann Surg Treat Res (2017) 93(3):130–6. doi: 10.4174/astr.2017.93.3.130 PMC5597536 28932728

[42] Bian SB Shen WS Xi HQ Wei B Chen L. Palliative therapy for gastric outlet obstruction caused by unresectable gastric cancer: A meta–analysis comparison of gastrojejunostomy with endoscopic stenting. Chin Med J (Engl) (2016) 129(9):1113–21. doi: 10.4103/0366-6999.180530 PMC4852681 27098799

[43] Ouchi K Sugawara T Ono H Fujiya T Kamiyama Y Kakugawa Y. Therapeutic significance of palliative operations for gastric cancer for survival and quality of life. J Surg Oncol (1998) 69(1):41–4. doi: 10.1002/(sici)1096-9098(199809)69:1<41::aid-jso8>3.0.co,2-k 9762890

[44] Prager LP Huff ML Alfieri SE Sujka JA. A fully stapled technique for gastrojejunal anastomosis creation in robotic Roux-en-Y gastric bypass. Langenbecks Arch Surg 407(8):3311–4. doi: 10.1007/s00423-022-02634-1 35927523

[45] Yao S Kita R Ishikawa K Kumode T Tanino K Mizuno R. Is palliative gastrojejunostomy for malignant gastric outlet obstruction an optimal choice for very elderly patients? A report from a long–lived country. Ann Surg Oncol (2023) 30(1):313–21. doi: 10.1245/s10434-022-12599-6 36171530

[46] Itoi T Baron TH Khashab MA Tsuchiya T Irani S Dhir V. Technical review of endoscopic ultrasonography–guided gastroenterostomy in 2017. Dig Endosc (2017) 29(4):495–502. doi: 10.1111/den.12794 28032663

[47] Chen YI Kunda R Storm AC Aridi HD Thompson CC Nieto J. EUS–guided gastroenterostomy: A multicenter study comparing the direct and balloon–assisted techniques. Gastrointest Endosc (2018) 87(5):1215–21. doi: 10.1016/j.gie.2017.07.030 28750837

[48] McCarty TR Garg R Thompson CC Rustagi T. Efficacy and safety of EUS–guided gastroenterostomy for benign and malignant gastric outlet obstruction: A systematic review and meta–analysis. Endosc Int Open (2019) 7(11):E1474–82. doi: 10.1055/a-0996-8178 PMC6811354 31673620

[49] Ojima T Nakamori M Nakamura M Katsuda M Hayata K Yamaue H. Laparoscopic gastrojejunostomy for patients with unresectable gastric cancer with gastric outlet obstruction. J Gastrointest Surg (2017) 21(8):1220–5. doi: 10.1007/s11605-017-3387-0 28224464

[50] Boghossian MB Funari MP De Moura DTH McCarty TR Sagae VMT Chen YI. EUS–guided gastroenterostomy versus duodenal stent placement and surgical gastrojejunostomy for the palliation of malignant gastric outlet obstruction: A systematic review and meta–analysis. Langenbecks Arch Surg (2021) 406(6):1803–17. doi: 10.1007/s00423-021-02215-8 34121130

[51] On W Huggett MT Young A Pine J Smith AM Tehami N. Endoscopic ultrasound guided gastrojejunostomy in the treatment of gastric outlet obstruction: multi-centre experience from the United Kingdom. Surg Endosc (2022). doi: 10.1007/s00464-022-09692-y PMC10017786 36217058

[52] Yoshida Y Fukutomi A Tanaka M Sugiura T Kawata N Kawai S. Gastrojejunostomy versus duodenal stent placement for gastric outlet obstruction in patients with unresectable pancreatic cancer. Pancreatology (2017) 17(6):983–9. doi: 10.1016/j.pan.2017.09.011 29066391

[53] Binmoeller KF Shah JN. Endoscopic ultrasound–guided gastroenterostomy using novel tools designed for transluminal therapy: A porcine study. Endoscopy (2012) 44(5):499–503. doi: 10.1055/s-0032-1309382 22531985

[54] Miyazaki Y Takiguchi S Takahashi T Kurokawa Y Makino T Yamasaki M. Treatment of gastric outlet obstruction that results from unresectable gastric cancer: Current evidence. World J Gastrointest Endosc (2016) 8(3):165–72. doi: 10.4253/wjge.v8.i3.165 PMC4734975 26862366

[55] Giordano S Victorzon M. Laparoscopic roux–En–Y gastric bypass in elderly patients (60 years or older): A meta–analysis of comparative studies. Scand J Surg (2018) 107(1):6–13. doi: 10.1177/1457496917731183 28942708

[56] Tyberg A Kats D Choi A Gaidhane M Nieto J Kahaleh M. Endoscopic ultrasound guided gastroenterostomy: What is the learning curve? J Clin Gastroenterol (2021) 55(8):691–3. doi: 10.1097/MCG.0000000000001400 32740096

[57] Lee HJ Hyung WJ Yang HK Han SU Park YK An JY. Short–term outcomes of a multicenter randomized controlled trial comparing laparoscopic distal gastrectomy with D2 lymphadenectomy to open distal gastrectomy for locally advanced gastric cancer (KLASS–02–RCT). Ann Surg (2019) 270(6):983–91. doi: 10.1097/SLA.0000000000003217 30829698

[58] Lee HJ Hyung WJ Yang HK Han SU Park YK An JY. Efficacy of endoscopic gastroduodenal stenting for gastric outlet obstruction due to unresectable advanced gastric cancer: A prospective multicenter study. J Surg Oncol (2014) 109(3):208–12. doi: 10.1002/jso.23486 24464867

[59] Potz BA Miner TJ. Surgical palliation of gastric outlet obstruction in advanced malignancy. World J Gastrointest Surg (2016) 8(8):545–55. doi: 10.4240/wjgs.v8.i8.545 PMC5003933 27648158

[60] Hong J Chen Y Li J Hu P Chen P Du N. Comparison of gastrojejunostomy to endoscopic stenting for gastric outlet obstruction: An updated systematic review and meta–analysis. Am J Surg (2022) 223(6):1067–78. doi: 10.1016/j.amjsurg.2021.10.038 34728070

[61] Sterpetti AV Fiori E Sapienza P Lamazza A. Complications after endoscopic stenting for malignant gastric outlet obstruction: A cohort study. Surg Laparosc Endosc Percutan Tech (2019) 29(3):169–72. doi: 10.1097/SLE.0000000000000656 30855401

[62] Khamar J Lee Y Sachdeva A Anpalagan T McKechnie T Eskicioglu C. Gastrojejunostomy versus endoscopic stenting for the palliation of malignant gastric outlet obstruction: A systematic review and meta–analysis. Surg Endosc (2022). doi: 10.1007/s00464-022-09572-5 36138247

[63] Haga Y Hiki N Kinoshita T Ojima T Nabeya Y Kuwabara S. Treatment option of endoscopic stent insertion or gastrojejunostomy for gastric outlet obstruction due to gastric cancer: A propensity score–matched analysis. Gastric Cancer (2020) 23(4):667–76. doi: 10.1007/s10120-020-01040-0 31982964

[64] Majumder S Buttar NS Gostout C Levy MJ Martin J Petersen B. Lumen–apposing covered self–expanding metal stent for management of benign gastrointestinal strictures. Endosc Int Open (2016) 4(1):E96–E101. doi: 10.1055/s-0041-108195 26793793 PMC4713186

[65] Kim JH Song HY Shin JH Hu HT Lee SK Jung HY. Metallic stent placement in the palliative treatment of malignant gastric outlet obstructions: Primary gastric carcinoma versus pancreatic carcinoma. AJR Am J Roentgenol (2009) 193(1):241–7. doi: 10.2214/AJR.08.1760 19542420

[66] Fiori E Crocetti D Sapienza P Cirocchi R Sterpetti AV Miccini M. Palliative surgery or metallic stent positioning for advanced gastric cancer: Differences in QOL. Med (Kaunas) (2021) 57(5):428. doi: 10.3390/medicina57050428 PMC8146574 33925171

[67] Krishnamoorthi R Bomman S Benias P Kozarek RA Peetermans JA McMullen E. Efficacy and safety of endoscopic duodenal stent versus endoscopic or surgical gastrojejunostomy to treat malignant gastric outlet obstruction: Systematic review and meta–analysis. Endosc Int Open (2022) 10(6):E874–97. doi: 10.1055/a-1794-0635 PMC9187371 35692924

[68] Park CH Park JC Kim EH Chung H An JY Kim HI. Impact of carcinomatosis and ascites status on long–term outcomes of palliative treatment for patients with gastric outlet obstruction caused by unresectable gastric cancer: Stent placement versus palliative gastrojejunostomy. Gastrointest Endosc (2015) 81(2):321–32. doi: 10.1016/j.gie.2014.06.024 25085332

[69] Laitamäki M Tyrväinen T Lehto JT Laukkarinen J Ukkonen M. Endoscopic duodenal stenting is efficient, but has higher rate of reoperations than gastrojejunostomy in palliative treatment for gastric outlet obstruction. Langenbecks Arch Surg (2022) 407(6):2509–15. doi: 10.1007/s00423-022-02565-x PMC9468122 35648229

[70] Jang SH Lee H Min BH Kim SM Kim HS Carriere KC. Palliative gastrojejunostomy versus endoscopic stent placement for gastric outlet obstruction in patients with unresectable gastric cancer: A propensity score–matched analysis. Surg Endosc (2017) 31(10):4217–23. doi: 10.1007/s00464-017-5480-6 28281127

[71] Dresner SM Wayman J Lovel T Hayes N Griffin SM. Percutaneous endoscopic duodenostomy: The relief of obstruction in advanced gastric carcinoma. Palliat Med (1999) 13(2):165–7. doi: 10.1191/026921699670950414 10474702

[72] Park KB Do YS Kang WK Choo SW Han YH Suh SW. Malignant obstruction of gastric outlet and duodenum: Palliation with flexible covered metallic stents. Radiology (2001) 219(3):679–83. doi: 10.1148/radiology.219.3.r01jn21679 11376254

[73] Jeurnink SM Steyerberg EW van Hooft JE van Eijck CH Schwartz MP Vleggaar FP. Surgical gastrojejunostomy or endoscopic stent placement for the palliation of malignant gastric outlet obstruction (SUSTENT study): A multicenter randomized trial. Gastrointest Endosc (2010) 71(3):490–9. doi: 10.1016/j.gie.2009.09.042 20003966

[74] Ohtsu A. Chemotherapy for metastatic gastric cancer: Past, present, and future. J Gastroenterol (2008) 43(4):256–64. doi: 10.1007/s00535-008-2177-6 18458840

[75] Smyth EC Verheij M Allum W Cunningham D Cervantes A Arnold D. Gastric cancer: ESMO clinical practice guidelines for diagnosis, treatment and follow–up. Ann Oncol (2016) 27(suppl 5):v38–v49. doi: 10.1093/annonc/mdw350 27664260

[76] Minami Y Kanemura S Oikawa T Suzuki S Hasegawa Y Miura K. Associations of cigarette smoking and alcohol drinking with stomach cancer survival: A prospective patient cohort study in Japan. Int J Cancer (2018) 143(5):1072–85. doi: 10.1002/ijc.31408 29603213

[77] Yang K Liu K Zhang WH Lu ZH Chen XZ Chen XL. The value of palliative gastrectomy for gastric cancer patients with intraoperatively proven peritoneal seeding. Med (Baltimore) (2015) 94(27):e1051. doi: 10.1097/MD.0000000000001051 PMC4504616 26166075

[78] Sakuramoto S Sasako M Yamaguchi T Kinoshita T Fujii M Nashimoto A. Adjuvant chemotherapy for gastric cancer with s–1, an oral fluoropyrimidine (published correction appears in n engl J med. N Engl J Med (2007) 357(18):1810–20. doi: 10.1056/NEJMoa072252 17978289

[79] Bang YJ Kim YW Yang HK Chung HC Park YK Lee KH. Adjuvant capecitabine and oxaliplatin for gastric cancer after D2 gastrectomy (CLASSIC): A phase 3 open–label, randomised controlled trial. Lancet (2012) 379(9813):315–21. doi: 10.1016/S0140-6736(11)61873-4 22226517

[80] Kim TH Ahn MS Choi YW Kang SY Choi JH Lee HW. Analysis of treatment outcomes according to the cycles of adjuvant chemotherapy in gastric cancer: A retrospective nationwide cohort study. BMC Cancer (2022) 22(1):948. doi: 10.1186/s12885-022-10006-7 36057562 PMC9440578

[81] Iwasaki Y Sasako M Yamamoto S Nakamura K Sano T Katai H. Phase II study of preoperative chemotherapy with s–1 and cisplatin followed by gastrectomy for clinically resectable type 4 and large type 3 gastric cancers (JCOG0210). J Surg Oncol (2013) 107(7):741–5. doi: 10.1002/jso.23301 23400787

[82] Zhu YJ Wu XY Wang W Chang XS Zhan DD Diao DC. Acupuncture for quality of life in gastric cancer patients undergoing adjuvant chemotherapy. J Pain Symptom Manage (2022) 63(2):210–20. doi: 10.1016/j.jpainsymman.2021.09.009 PMC11488290 34563627

[83] Wang Z Chen JQ Liu JL Tian L. Issues on peritoneal metastasis of gastric cancer: An update. World J Surg Oncol (2019) 17(1):215. doi: 10.1186/s12957-019-1761-y 31829265 PMC6907197

[84] Grass F Vuagniaux A Teixeira–Farinha H Lehmann K Demartines N Hübner M. Systematic review of pressurized intraperitoneal aerosol chemotherapy for the treatment of advanced peritoneal carcinomatosis. Br J Surg (2017) 104(6):669–78. doi: 10.1002/bjs.10521 28407227

[85] Kobayashi D Kodera Y. Intraperitoneal chemotherapy for gastric cancer with peritoneal metastasis. Gastric Cancer (2017) 20(Suppl 1):111–21. doi: 10.1007/s10120-016-0662-9 27803990

[86] Horvath P Yurttas C Baur I Steidle C Reymond MA Girotti PNC. Current medical care situation of patients in Germany undergoing pressurized intraperitoneal aerosol chemotherapy (PIPAC). Cancers (Basel) (2022) 14(6):1443. doi: 10.3390/cancers14061443 35326595 PMC8946267

[87] Alyami M Bonnot PE Mercier F Laplace N Villeneuve L Passot G. Pressurized intraperitoneal aerosol chemotherapy (PIPAC) for unresectable peritoneal metastasis from gastric cancer. Eur J Surg Oncol (2021) 47(1):123–7. doi: 10.1016/j.ejso.2020.05.021 32561204

[88] Li Z Wong LCK Sultana R Lim HJ Tan JW Tan QX. A systematic review on quality of life (QoL) of patients with peritoneal metastasis (PM) who underwent pressurized intraperitoneal aerosol chemotherapy (PIPAC). Pleura Peritoneum (2022) 7(2):39–49. doi: 10.1515/pp-2021-0154 35812010 PMC9166188

[89] Kim HS Kim JH Kim JW Kim BC. Chemotherapy in elderly patients with gastric cancer. J Cancer (2016) 7(1):88–94. doi: 10.7150/jca.13248 26722364 PMC4679385

[90] He S Shen J Hong L Niu L Niu D. Capecitabine "metronomic" chemotherapy for palliative treatment of elderly patients with advanced gastric cancer after fluoropyrimidine–based chemotherapy. Med Oncol (2012) 29(1):100–6. doi: 10.1007/s12032-010-9791-x 21264547

[91] Liao PW Cheng SB Chou CW Lin HC Lin CH Chen TC. Chemotherapy for metastatic gastric cancer: Does age matter? A single–center, retrospective, real–world study. Clin Med Insights Oncol (2022) 16:11795549221123617. doi: 10.1177/11795549221123617 36134036 PMC9483662

[92] Cainap C Vlad C Seicean A Balacescu O Seicean R Constantin AM. Gastric cancer: Adjuvant chemotherapy versus chemoradiation. A clinical point of view. J BUON (2019) 24(6):2209–19. 31983085

[93] Coccolini F Fugazzola P Ansaloni L Sartelli M Cicuttin E Leandro G. Advanced gastric cancer: The value of systemic and intraperitoneal chemotherapy. Acta BioMed (2018) 89(8–S):104–9. doi: 10.23750/abm.v89i8-S.7904 PMC6502214 30561427

[94] Zurleni T Gjoni E Altomare M Rausei S. Conversion surgery for gastric cancer patients: A review. World J Gastrointest Oncol (2018) 10(11):398–409. doi: 10.4251/wjgo.v10.i11.398 30487951 PMC6247102

[95] Shchepotin IB Chorny V Hanfelt J Evans SR. Palliative superselective intra–arterial chemotherapy for advanced nonresectable gastric cancer. J Gastrointest Surg (1999) 3(4):426–31. doi: 10.1016/s1091-255x(99)80060-2 10482696

[96] Bang YJ Van Cutsem E Feyereislova A Chung HC Shen L Sawaki A. Trastuzumab in combination with chemotherapy versus chemotherapy alone for treatment of HER2–positive advanced gastric or gastro–oesophageal junction cancer (ToGA): A phase 3, open–label, randomised controlled trial (published correction appears in lancet. Lancet (2010) 376(9742):687–97. doi: 10.1016/S0140-6736(10)61121-X 20728210

[97] Li XD Shen H Jiang JT Zhang HZ Zheng X Shu YQ. Paclitaxel based vs oxaliplatin based regimens for advanced gastric cancer. World J Gastroenterol (2011) 17(8):1082–7. doi: 10.3748/wjg.v17.i8.1082 PMC3057154 21448363

[98] Ajani JA Buyse M Lichinitser M Gorbunova V Bodoky G Douillard JY. Combination of cisplatin/S–1 in the treatment of patients with advanced gastric or gastroesophageal adenocarcinoma: Results of noninferiority and safety analyses compared with cisplatin/5–fluorouracil in the first–line advanced gastric cancer study. Eur J Cancer (2013) 49(17):3616–24. doi: 10.1016/j.ejca.2013.07.003 23899532

[99] Dong L Li J Lou XP Miao JH Lu P Chang ZW. Comparison of short–term efficacy and safety of TIROX and DCF regimens for advanced gastric cancer. J Int Med Res (2014) 42(3):737–43. doi: 10.1177/0300060513510657 PMC8280548 24717407

[100] Ochenduszko S Puskulluoglu M Konopka K Fijorek K Urbanczyk K Budzynski A. Comparison of efficacy and safety of first–line palliative chemotherapy with EOX and mDCF regimens in patients with locally advanced inoperable or metastatic HER2–negative gastric or gastroesophageal junction adenocarcinoma: A randomized phase 3 trial. Med Oncol (2015) 32(10):242. doi: 10.1007/s12032-015-0687-7 26354521 PMC4564435

[101] Quintero–Aldana G Jorge M Grande C Salgado M Gallardo E Varela S. Phase II study of first–line biweekly docetaxel and cisplatin combination chemotherapy in advanced gastric cancer. Cancer Chemother Pharmacol (2015) 76(4):731–7. doi: 10.1007/s00280-015-2839-z 26242221

[102] Yamada Y Higuchi K Nishikawa K Gotoh M Fuse N Sugimoto N. Phase III study comparing oxaliplatin plus s–1 with cisplatin plus s–1 in chemotherapy–naïve patients with advanced gastric cancer. Ann Oncol (2015) 26(1):141–8. doi: 10.1093/annonc/mdu472 25316259

[103] Chen X Li W Sun L Liu Y Liu S Ma R. S–l combined with cisplatin plus concurrent chemoradiotherapy versus cisplatin plus concurrent chemoradiotherapy for Chinese patients with advanced gastric cancer: A multi–centre randomized controlled trial. Clin Transl Oncol (2016) 18(7):672–6. doi: 10.1007/s12094-015-1416-6 26482723

[104] Wang J Xu R Li J Bai Y Liu T Jiao S. Randomized multicenter phase III study of a modified docetaxel and cisplatin plus fluorouracil regimen compared with cisplatin and fluorouracil as first–line therapy for advanced or locally recurrent gastric cancer. Gastric Cancer (2016) 19(1):234–44. doi: 10.1007/s10120-015-0457-4 PMC4688303 25604851

[105] Fushida S Kinoshita J Kaji M Oyama K Hirono Y Tsukada T. Paclitaxel plus valproic acid versus paclitaxel alone as second– or third–line therapy for advanced gastric cancer: A randomized phase II trial. Drug Des Devel Ther (2016) 10:2353–8. doi: 10.2147/DDDT.S110425 PMC4966651 27524882

[106] Sah BK Zhang B Zhang H Li J Yuan F Ma T. Neoadjuvant FLOT versus SOX phase II randomized clinical trial for patients with locally advanced gastric cancer. Nat Commun (2020) 11(1):6093. doi: 10.1038/s41467-020-19965-6 33257672 PMC7705676

[107] Kawakami H Fujitani K Matsuyama J Akamaru Y Tamura S Endo S. Comparison of s–1–cisplatin every 5 weeks with capecitabine–cisplatin every 3 weeks for HER2–negative gastric cancer (recurrent after s–1 adjuvant therapy or chemotherapy–naïve advanced): Pooled analysis of HERBIS–2 (OGSG 1103) and HERBIS–4A (OGSG 1105) trials. Int J Clin Oncol (2020) 25(9):1635–43. doi: 10.1007/s10147-020-01711-z 32494981

[108] Zhu XD Huang MZ Wang YS Feng WJ Chen ZY He YF. XELOX doublet regimen versus EOX triplet regimen as first–line treatment for advanced gastric cancer: An open–labeled, multicenter, randomized, prospective phase III trial (EXELOX). Cancer Commun (Lond) (2022) 42(4):314–26. doi: 10.1002/cac2.12278 PMC9017757 35212487

[109] Bin Y Lan D Bao W Yang H Zhou S Huang F. SOX combined with intraperitoneal perfusion of docetaxel compared with DOS regimen in the first–line therapy for advanced gastric cancer with malignant ascites: A prospective observation. Trials (2022) 23(1):211. doi: 10.1186/s13063-022-06143-w 35279214 PMC8917651

[110] Rosati G Cella CA Cavanna L Codecà C Prisciandaro M Mosconi S. A randomized phase III study of fractionated docetaxel, oxaliplatin, capecitabine (low–tox) vs epirubicin, oxaliplatin and capecitabine (eox) in patients with locally advanced unresectable or metastatic gastric cancer: The lega trial. Gastric Cancer (2022) 25(4):783–93. doi: 10.1007/s10120-022-01292-y 35352176

[111] Williams GR Manjunath SH Butala AA Jones JA. Palliative radiotherapy for advanced cancers: Indications and outcomes. Surg Oncol Clin N Am (2021) 30(3):563–80. doi: 10.1016/j.soc.2021.02.007 34053669

[112] Tey J Choo BA Leong CN Loy EY Wong LC Lim K. Clinical outcome of palliative radiotherapy for locally advanced symptomatic gastric cancer in the modern era. Med (Baltimore) (2014) 93(22):e118. doi: 10.1097/MD.0000000000000118 PMC4616316 25396330

[113] Tey J Soon YY Koh WY Leong CN Choo BA Ho F. Palliative radiotherapy for gastric cancer: A systematic review and meta–analysis. Oncotarget (2017) 8(15):25797–805. doi: 10.18632/oncotarget.15554 PMC5421969 28445941

[114] Kondoh C Shitara K Nomura M Takahari D Ura T Tachibana H. Efficacy of palliative radiotherapy for gastric bleeding in patients with unresectable advanced gastric cancer: A retrospective cohort study. BMC Palliat Care (2015) 14:37. doi: 10.1186/s12904-015-0034-y 26238344 PMC4524128

[115] Takeda K Sakayauchi T Kubozono M Katagiri Y Umezawa R Yamamoto T. Palliative radiotherapy for gastric cancer bleeding: A multi–institutional retrospective study. BMC Palliat Care (2022) 21(1):52. doi: 10.1186/s12904-022-00943-2 35413824 PMC9004196

[116] Saito T Kosugi T Nakamura N Wada H Tonari A Ogawa H. Treatment response after palliative radiotherapy for bleeding gastric cancer: A multicenter prospective observational study (JROSG 17–3). Gastric Cancer (2022) 25(2):411–21. doi: 10.1007/s10120-021-01254-w 34580795

[117] Tey J Zheng H Soon YY. Palliative radiotherapy in symptomatic locally advanced gastric cancer: A phase II trial. Cancer Med (2019) 8(4):1447–58. doi: 10.1002/cam4.2021 PMC6488108 30790469

[118] Kawabata H Uno K Yasuda K Yamashita M. Experience of low–dose, short–course palliative radiotherapy for bleeding from unresectable gastric cancer. J Palliat Med (2017) 20(2):177–80. doi: 10.1089/jpm.2016.0141 27672720

[119] Asakura H Hashimoto T Harada H Mizumoto M Furutani K Hasuike N. Palliative radiotherapy in symptomatic locally advanced gastric cancer: A phase II trial. Cancer Med (2019) 8(4):1447–58. doi: 10.1002/cam4.2021 PMC6488108 30790469

[120] Hiramoto S Kikuchi A Tetsuso H Yoshioka A Kohigashi Y Maeda I. Efficacy of palliative radiotherapy and chemo–radiotherapy for unresectable gastric cancer demonstrating bleeding and obstruction. Int J Clin Oncol (2018) 23(6):1090–4. doi: 10.1007/s10147-018-1317-0 30019177

[121] Shao ZY Zhang JD. (2008). Zhonghua Yi Xue Za Zhi. 88(36):2547–9. 19080647

[122] Yoshikawa T Tsuburaya A Hirabayashi N Yoshida K Nagata N Kodera Y. A phase I study of palliative chemoradiation therapy with paclitaxel and cisplatin for local symptoms due to an unresectable primary advanced or locally recurrent gastric adenocarcinoma. Cancer Chemother Pharmacol (2009) 64(6):1071–7. doi: 10.1007/s00280-009-0963-3 19283353

[123] Kwon HC Kim MC Kim KH Jang JS Oh SY Kim SH. Adjuvant chemoradiation versus chemotherapy in completely resected advanced gastric cancer with D2 nodal dissection. Asia Pac J Clin Oncol (2010) 6(4):278–85. doi: 10.1111/j.1743-7563.2010.01331.x 21114777

[124] An JY Kim HI Cheong JH Hyung WJ Kim CB Noh SH. Pathologic and oncologic outcomes in locally advanced gastric cancer with neoadjuvant chemotherapy or chemoradiotherapy. Yonsei Med J (2013) 54(4):888–94. doi: 10.3349/ymj.2013.54.4.888 PMC3663211 23709422

[125] Boda–Heggemann J Weiss C Schneider V Hofheinz RD Haneder S Michaely H. Adjuvant IMRT/XELOX radiochemotherapy improves long–term overall– and disease–free survival in advanced gastric cancer. Strahlenther Onkol (2013) 189(5):417–23. doi: 10.1007/s00066-013-0309-2 23558673

[126] Nishikawa K Tsuburaya A Yoshikawa T Kobayashi M Kawada J Fukushima R. A randomised phase II trial of capecitabine plus cisplatin versus s–1 plus cisplatin as a first–line treatment for advanced gastric cancer: Capecitabine plus cisplatin ascertainment versus s–1 plus cisplatin randomised PII trial (XParTS II). Eur J Cancer (2018) 101:220–8. doi: 10.1016/j.ejca.2018.06.026 30096702

[127] Wang F Qu A Sun Y Zhang J Wei B Cui Y. Neoadjuvant chemoradiotherapy plus postoperative adjuvant XELOX chemotherapy versus postoperative adjuvant chemotherapy with XELOX regimen for local advanced gastric cancer–a randomized, controlled study. Br J Radiol (2021) 94(1124):20201088. doi: 10.1259/bjr.20201088 34260297 PMC8764914

